# A Novel Neural Substrate for the Transformation of Olfactory Inputs into Motor Output

**DOI:** 10.1371/journal.pbio.1000567

**Published:** 2010-12-21

**Authors:** Dominique Derjean, Aimen Moussaddy, Elias Atallah, Melissa St-Pierre, François Auclair, Steven Chang, Xiang Ren, Barbara Zielinski, Réjean Dubuc

**Affiliations:** 1 Department of Kinesiology, Université du Québec à Montréal, Montréal, Québec, Canada; 2 Groupe de Recherche sur le Système Nerveux Central, Department of Physiology, Université de Montréal, Montréal, Québec, Canada; 3 Department of Biological Sciences, University of Windsor, Windsor, Ontario, Canada; Brandeis, United States of America

## Abstract

It is widely recognized that animals respond to odors by generating or modulating specific motor behaviors. These reactions are important for daily activities, reproduction, and survival. In the sea lamprey, mating occurs after ovulated females are attracted to spawning sites by male sex pheromones. The ubiquity and reliability of olfactory-motor behavioral responses in vertebrates suggest tight coupling between the olfactory system and brain areas controlling movements. However, the circuitry and the underlying cellular neural mechanisms remain largely unknown. Using lamprey brain preparations, and electrophysiology, calcium imaging, and tract tracing experiments, we describe the neural substrate responsible for transforming an olfactory input into a locomotor output. We found that olfactory stimulation with naturally occurring odors and pheromones induced large excitatory responses in reticulospinal cells, the command neurons for locomotion. We have also identified the anatomy and physiology of this circuit. The olfactory input was relayed in the medial part of the olfactory bulb, in the posterior tuberculum, in the mesencephalic locomotor region, to finally reach reticulospinal cells in the hindbrain. Activation of this olfactory-motor pathway generated rhythmic ventral root discharges and swimming movements. Our study bridges the gap between behavior and cellular neural mechanisms in vertebrates, identifying a specific subsystem within the CNS, dedicated to producing motor responses to olfactory inputs.

## Abstract 2

Anatomical and physiological experiments in the lamprey reveal the neural circuit involved in transforming olfactory inputs into motor outputs, which was previously unknown in a vertebrate.

## Author Summary

Animal behaviors, including locomotion, can be driven by olfactory cues, such as pheromones or food sources. The neural substrate (neuroanatomical connections and physiological signals) that permits the transformation of olfactory inputs into locomotor responses is still unknown in vertebrates. In the present study, we identify such a neural substrate in the lamprey. Here, olfactory signals from the outside world are transmitted to the reticulospinal neurons in the lower brainstem, which provide the descending locomotor command to the spinal cord. We found that this circuit originates in the medial portion of the olfactory bulb and that connections are made in the posterior tuberculum, a ventral diencephalic structure. These inputs are then conveyed to the mesencephalic locomotor region, known to project extensively to brainstem reticulospinal neurons and thereby activate locomotion. Our results illuminate a specific dedicated neural substrate in the brain of lampreys that underlies olfactory-motor responses, which is activated by both food-related or pheromonal olfactory cues. It will be of interest to determine whether such a pathway is preserved in all vertebrates.

## Introduction

Animals use olfaction in different behavioral contexts such as food seeking, social communication, and reproduction. Motor behaviors, including locomotion, can be generated by olfactory stimulation in several species of vertebrates, including fishes [1], rats [2], humans [3], and lampreys [4]. The highly conserved and stereotyped nature of some of those motor responses suggests a strong neural link between olfactory inputs and motor command centers in the central nervous system (CNS). The neural pathways and the underlying mechanisms responsible for olfactory-motor behavior have only been revealed in one invertebrate species [5]. Here we show the neural substrates for olfactory-motor transformations in vertebrates by using an in vitro lamprey preparation.

It is well established that parasitic sea lampreys increase their locomotor activity towards prey odors [6]. Later in life, pheromones direct movement during upstream migration [7],[8] and spawning [4],[9]. Fifty-nine chemosensory genes representing three gene families have been identified in the peripheral olfactory organ of the sea lamprey [10], a structure containing the main olfactory epithelium, as well as an adjacent accessory olfactory organ. Accessory olfactory neurons project solely to glomerular neuropil in the medial olfactory bulb (OB), while main olfactory neurons project to both medial and non-medial glomerular neuropil [11]. The GTP binding protein G_olf_ is localized in the sensory neurons located in the main olfactory epithelium projecting to the non-medial OB glomeruli but is absent from olfactory sensory neurons that project into the medial region of the OB [12]. In the sea lamprey, basic amino acids and bile acids, as well as reproductive and migratory pheromones, elicit robust field potential responses in the peripheral olfactory organ [8],[13]–[15]. While the anatomy of the lamprey olfactory system, including the OB projections to the telencephalon and diencephalon, has been partly described [16], the locomotor control system of lampreys is far better understood. Reticulospinal (RS) cells directly activate the spinal locomotor networks [17],[18] and act as command neurons for generating locomotor movements. The RS cells receive inputs from the periphery, the spinal cord, and locomotor centers in the forebrain and brainstem, including the mesencephalic locomotor region (MLR [19]–[21]), a highly conserved neural center controlling locomotion in all vertebrate species. Another locomotor center located in the diencephalon has been described in lampreys [22]. This region, referred to as the diencephalic locomotor region (DLR), also projects directly to RS cells. The exact contribution of MLR and DLR to locomotor control has not been established yet, but we know that RS cell activation is a prerequisite for movement production. The activity of these cells constitutes an excellent monitor for motor activation in lampreys. We took advantage of this and developed a preparation in which the brain and rostral spinal cord were isolated in vitro, along with an intact olfactory epithelium (see the Materials and Methods section). We applied physiological and anatomical tools to examine the neural substrate of olfactory-motor responses in this preparation, which provides excellent accessibility to all potential relays carrying olfactory inputs to motor command neurons in the brainstem. To confirm the locomotor role of potential relay areas of olfactory inputs, we used a semi-intact preparation, with the tail left attached to monitor swimming behavior.

## Results

### Activation of RS Neurons by Olfactory Inputs

**Figure 1:**
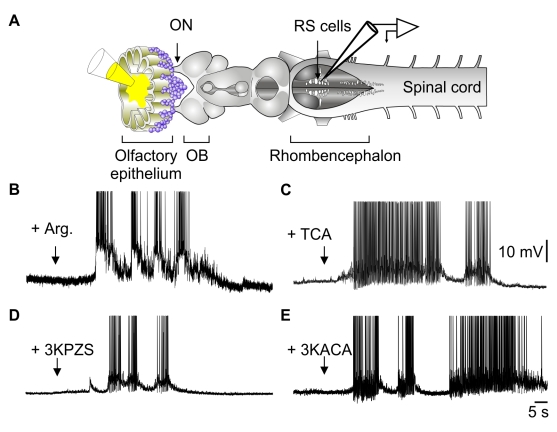
Olfactory epithelium stimulation activates RS cells. (A) Illustration of the experimental procedure in an isolated olfactory epithelium-brain-spinal cord preparation. (B) Responses of RS cell to the application of L-arginine over the olfactory epithelium (Arg, 1 mM). (C) Response to bile acid–taurocholic acid (TCA, 1 µM). (D–E) Responses to male-secreted pheromones, 3-keto-petromyzonol sulfate (3KPZS, 10 µM), and 3-keto allocholic acid (3KACA, 10 µM), respectively. Arrows represent the onset of odor ejection. (B–E) are from different preparations.

We used an isolated brain-spinal cord preparation, with an intact olfactory epithelium, to examine the effect of odor on the responses of RS cells recorded intracellularly. We found that odor stimulation of the olfactory sensory neurons induced large excitatory responses in RS cells. Lamprey pheromones have already been demonstrated to attract adult lampreys and generate locomotion in their natural surroundings [4],[9]. Application of the odor molecules L-arginine (1 mM, *n* = 11, Figure 1B), the bile acid–taurocholic acid (1 µM, *n* = 3, Figure 1C), or the male-secreted pheromones 3-keto petromyzonol sulfate (3KPZS; 10 µM, *n* = 3, Figure 1D) and 3-keto allocholic acid (3KACA, 10 µM, *n* = 3, Figure 1E) onto the olfactory epithelium using a Picospritzer injection system induced large sustained depolarizations with spiking activity in RS cells. The excitatory responses occurred on average 35.7±6 s after the application, with similar latencies for all olfactory substances. These long latencies likely reflected the time needed for the odor molecules to diffuse into the lumen of the peripheral olfactory organ and reach olfactory sensory neurons. Excitatory responses to odors and pheromones were observed in RS cells of both females and males. Application of the Ringer's carrier solution failed to elicit responses. It is noteworthy that often we did not get any response in preparations from these wild-caught animals, when kept in captivity for a long period of time. Desensitization of the olfactory sensory neurons or a modulatory depression within the pathway could have occurred, and further experiments are needed to clarify this question.

**Figure 2:**
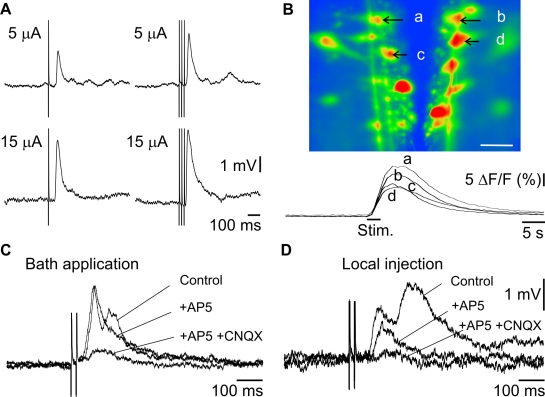
Olfactory nerve stimulation activates RS cells. (A) Responses of RS cells following electrical stimulation of the ON with 5 or 15 µA (top versus bottom traces); single shocks or trains of stimulation (left versus right traces). Each trace is a mean of eight individual responses. (B) Calcium fluorescence imaging illustrates the ΔF/F response of identified RS cells to ON stimulation (20 µA –10 Hz). (a, c) ipsilateral. (b, d) contralateral. White scale bar in the photomicrograph represents 100 µm. (C) RS responses to ON electrical stimulation are reduced by glutamate antagonists perfused through the bath (50 µA stimulation, upper traces) (D) or injected onto the OB (50 µA stimulation, bottom traces).

To investigate the physiological pathway involved, we first established whether RS cells responded to electrical stimulation of the olfactory nerve (ON). We stimulated the ON unilaterally and recorded synaptic responses in RS cells intracellularly (Figure 2A). The RS cells on both sides displayed excitatory responses, with a latency of 95±10 ms at the ipsilateral (*n* = 101) and 118±13 ms at the contralateral side (*n* = 44, not statistically different, Mann-Whitney test, *p* = 0.88). The constant latency with repeated stimulation and the sharp onset of the RS cell responses suggested a strong link between olfactory sensory areas and locomotor command neurons. Temporal summation was also observed when using trains of stimuli (Figure 2A), suggesting at least a few synapses along this pathway. Similar responses were seen from larvae, newly transformed, parasitic, and spawning animals (thus at different ages), suggesting the presence of anatomical connections between the olfactory and motor systems throughout the lamprey life cycle. Because the lamprey brainstem contains about 2,500 RS neurons, and intracellular recordings allow the sampling of a relatively limited number of them, we performed calcium imaging experiments to determine whether olfactory inputs also activated populations of RS cells. Repetitive stimulation of the ON (10–100 µA, 10 Hz, 2–10 s) elicited a large rise in intracellular calcium in many RS cells in the middle rhombencephalic reticular nucleus (MRRN) on both sides, in the six preparations examined (Figure 2B). The calcium responses lasted tens of seconds, which has been associated with lasting afterdischarges in RS cells [17].

**Figure 3:**
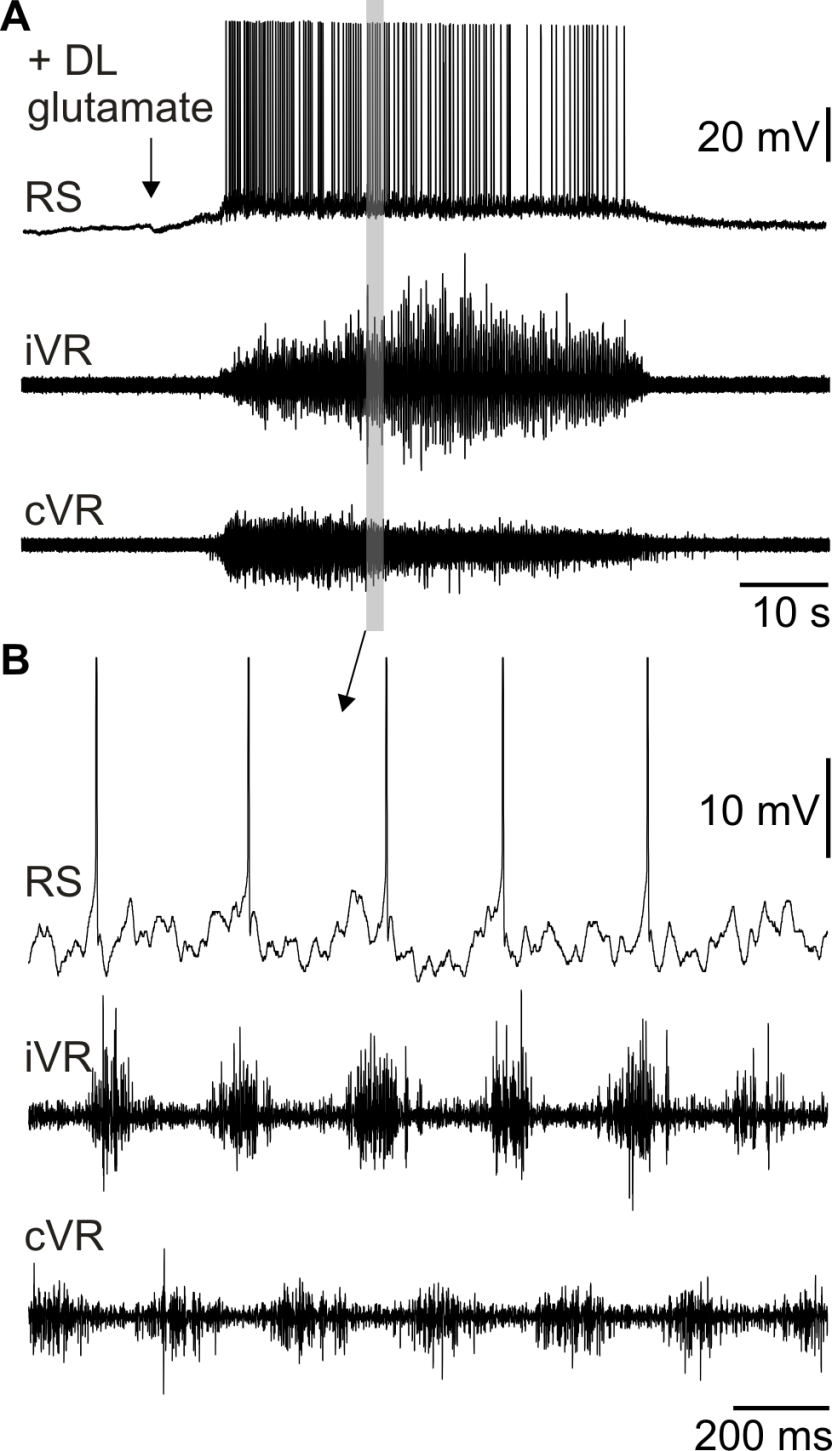
Glutamate injection into the OB induces fictive locomotion. (A) Top trace: Intracellular recording of a RS cell. Note the large excitation induced by the injection of 3 mM glutamate in the ipsilateral OB. Bottom traces: Ventral root (VR) discharges on both sides. (B) Detail from the boxed area in (B) shows fictive locomotion characterized by alternating ipsilateral and contralateral ventral root activity (iVR and cVR, respectively). Note that the RS cell shows rhythmic oscillations in tune with the fictive locomotor pattern.

Perfusion of glutamate receptor antagonists (AP5: 100 µM, CNQX: 10 µM, *n* = 3) into the recording chamber blocked the response of RS cells to ON stimulation (Figure 2C). In addition, local injections of these antagonists into the OB (AP5: 10 mM, CNQX: 1 mM, *n* = 6, Figure 2D) had the same effect, indicating that olfactory primary afferent inputs in the OB rely on glutamate neurotransmission. To determine whether the powerful excitation of RS cells produced by olfactory inputs was associated with locomotion, suction electrodes were positioned over the ventral roots to record the motor output generated by spinal cord networks. A unilateral local application of glutamate (3 mM, *n* = 4) in the OB induced large sustained depolarizations with superimposed action potentials in RS cells, while rhythmic bursts of activity alternated bilaterally in the ventral roots (Figure 3A–B). This type of motor activity is referred to as “fictive locomotion” [23]–[25]. Taken together, these experiments suggested a strong link between olfactory inputs and the neural control circuitry for locomotion.

### Localization of an OB Relay

**Figure 4:**
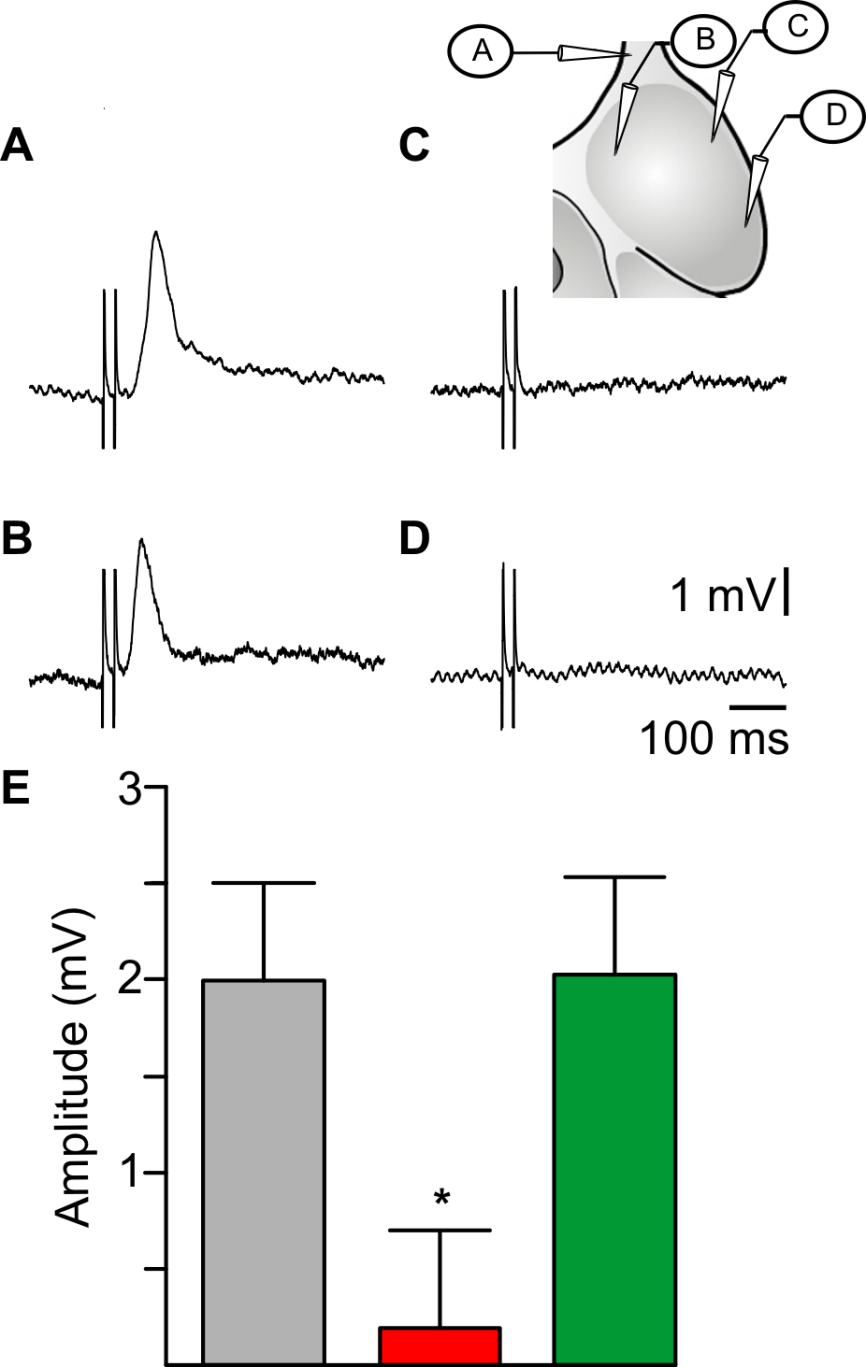
Olfactory-locomotor information transits through the medial region of the OB. (A–D) Responses in a single ipsilateral RS neuron to 30 µA stimulation of the ON and OB. The schematic (inset) indicates the location of stimulating electrodes. Note that a synaptic response was elicited only following stimulation of the ON or the medial part of the OB. (E) Mean amplitude of 4 RS cells responses to 30 µA ON stimulation before (grey bar) and after local injection of AP5 and CNQX mixture in the central-medial OB (red bar) and lateral OB (green bar). * *p*<0.05.

We then carried out experiments to physiologically identify the neural connectivity linking the olfactory input to locomotor centers. We first examined whether all OB regions transmitted olfactory inputs to RS cells, by comparing RS responses to ON stimulation, to those elicited by stimulating different parts of the OB (Figure 4A–D). Stimulating the medial part of the OB (30 µA) elicited excitatory synaptic responses in RS cells with a mean response amplitude of 2.1±0.3 mV (*n* = 13 animals, Figure 4B). Overall, RS responses to ipsilateral medial OB stimulation occurred with a mean latency of 71±7 ms (*n* = 39). Responses to 30 µA stimulation of the lateral portion of OB were much smaller (0.6±0.2 mV, paired *t* test, *p*<0.001) and often absent (Figure 4C, D). The threshold intensity needed for eliciting excitatory responses in RS cells was compared for stimulation of the ON and different parts of OB (not shown). Stimulating the ON or the medial part of the OB elicited RS responses with a similar mean threshold intensity of 4.2±1 µA and 12.4±5 µA, respectively (paired *t* test, *p* = 0.197; *n* = 5), whereas stimulation anywhere else in the OB required much stronger stimulation intensities. We tested stimulation intensities up to 50 µA in non-medial OB locations and RS responses were often absent. If responses were present, they never exceeded 1 mV in amplitude. The above results were confirmed by locally injecting glutamate receptor antagonists into the OB (AP5: 10 mM, CNQX: 1 mM). Only injections into the medial territories reduced the RS responses to ON stimulation (*n* = 4, Figure 4E). In addition, local injections of glutamate (3 mM) within the medial OB induced ventral root discharges, whereas injections in other parts had no effect (*n* = 3, not shown). RS synaptic responses to ON stimulation were not affected by surgical ablation of the lateral portion of the OB (*n* = 4, not shown). Altogether, these experiments indicate that the medial part of the OB is crucial for eliciting excitatory responses in RS cells.

### Neural Pathway Involved in Olfactory-Locomotor Transformations

**Figure 5:**
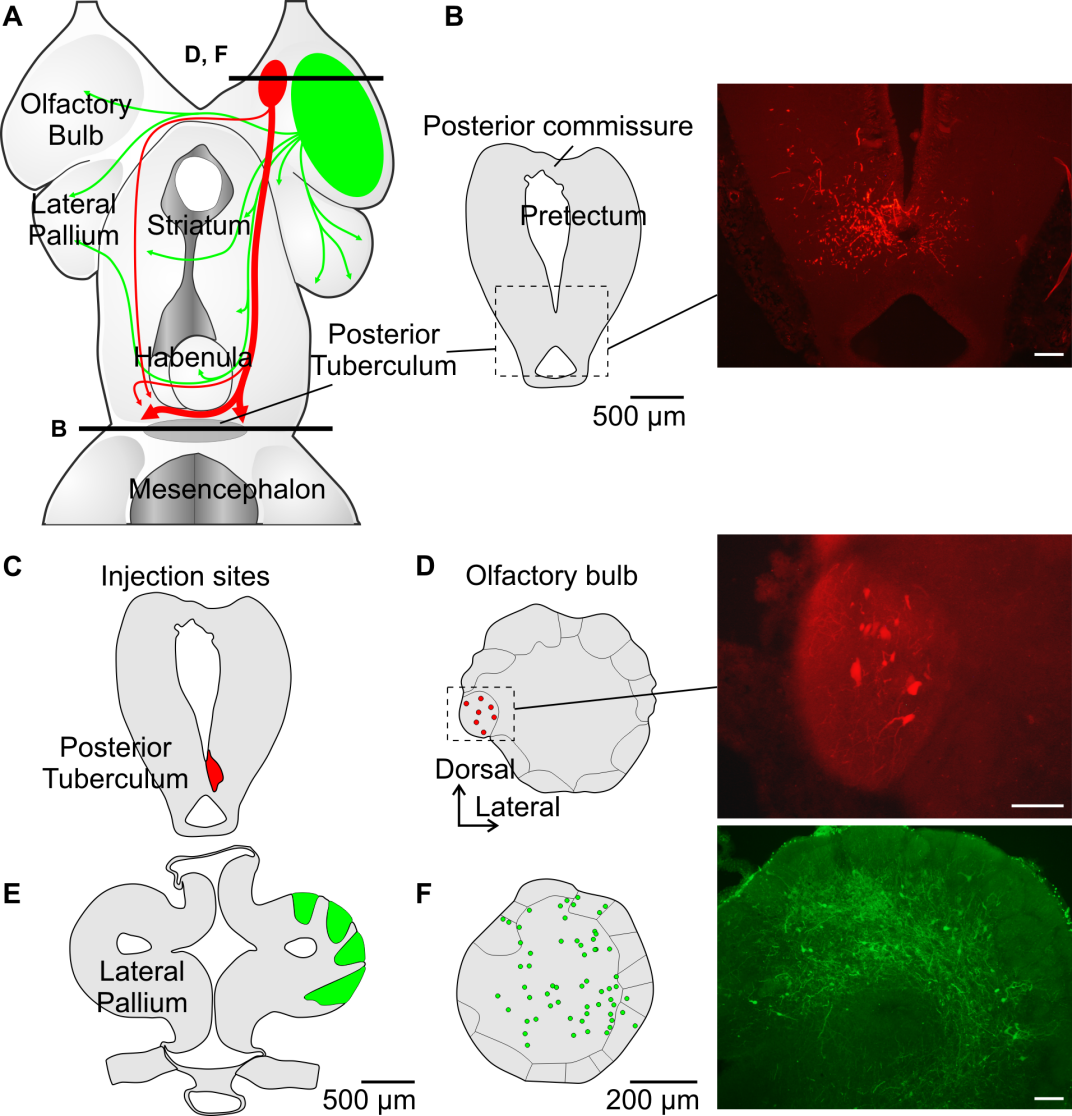
The medial region of the OB projects to the PT. (A) Schematic dorsal view of the forebrain summarizing the efferent OB projections in the lamprey. Projections from OB regions other than the medial region are shown in green. (B) Anterograde labeling from the medial OB (red) shows fibers terminating in the PT (see picture to the right). (C, D) Retrograde labeling from the PT shows neuronal cell bodies in only one medial glomerulus in the OB (see picture to the right). (E, F) Retrograde labeling from the lateral pallium shows neurons associated with almost all glomeruli, except the medial. White scale bars in pictures represent 100 µm.

Anatomical experiments were then carried out to determine whether the projections from the medial part of the OB differed from the rest of the OB. The lateral OB was injected with Texas Red-conjugated dextran amines (*n* = 10, Figure 5A) and anterograde labeling confirmed projections to the lateral pallium [11]. When the tracer was injected in the medial OB (*n* = 15, Figure 5A, B), labeled fibers were found descending bilaterally to a caudal ventral region of the diencephalon, the posterior tuberculum (PT). This region was previously shown to project to the MLR in lampreys [26]. We then carried out retrograde tracing experiments. Dorsal, lateral, and ventral injections into the lateral pallium (Figure 5A, E) labeled mitral-like cells throughout the dorsal (*n* = 7), lateral (*n* = 5), and ventral (*n* = 3) parts of the OB, respectively. No labeling was found in the medial OB region (Figure 5F). Interestingly, the OB labeling pattern following injection into the PT was altogether very different (Figure 5A, C, D). Retrogradely labeled neurons were found only in a medial OB glomerulus on both sides of the brain (*n* = 5, Figure 5C, D). The axonal projections were also examined anatomically. Two separate bundles left the OB—one coursed dorsally under the dorsomedial telencephalic neuropil and the other ventromedially through the septal and preoptic area. These two bundles merged into a single diffuse tract at the level of the caudal part of the dorsal pallium, extended caudally through the thalamus and hypothalamus, and terminated in the PT, where some fibers crossed the midline (see Figure 5A). These results indicated that there is a distinct projection from the medial part of the OB to the caudal ventral diencephalon that could elicit excitatory responses in RS cells and locomotor activity in response to glutamatergic excitation of the OB.

**Figure 6:**
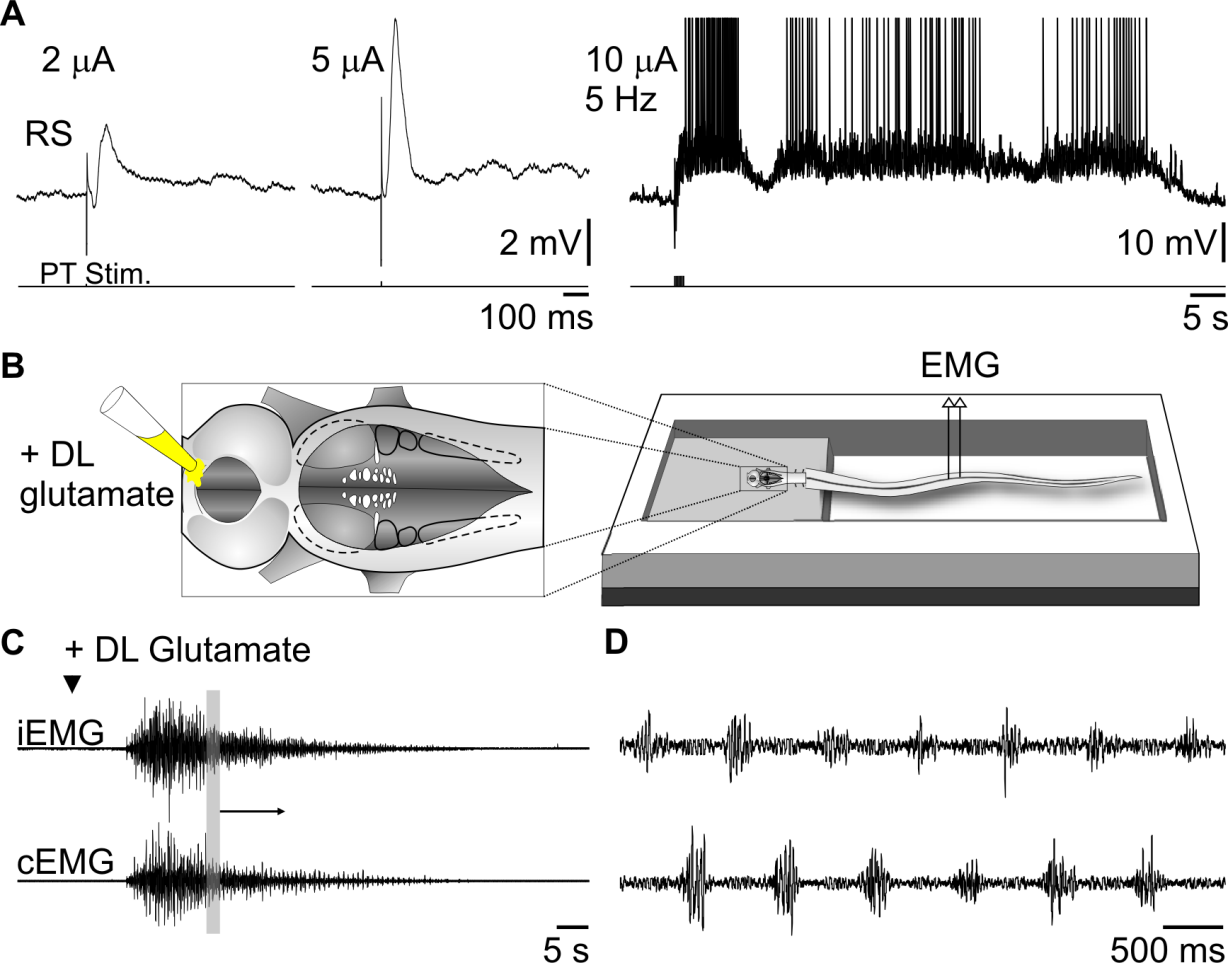
Stimulation of the PT activates RS neurons and locomotion. (A) PT stimulation induces RS responses. Raising the stimulation intensity from 2 to 5 µA increases the amplitude of the evoked synaptic responses (left two traces), while a short stimulation train (10 µA –5 Hz) elicited a long-lasting afterdischarge in the same RS cell (right trace). (B) Illustration of the semi-intact preparation. (C) Glutamate (3 mM) injected into the PT elicited swimming and bursts of activity on EMG recordings. iEMG, ipsilateral; cEMG, contralateral. (D) Enlargement of the boxed area in (C) shows left and right muscle contractions.

**Figure 7:**
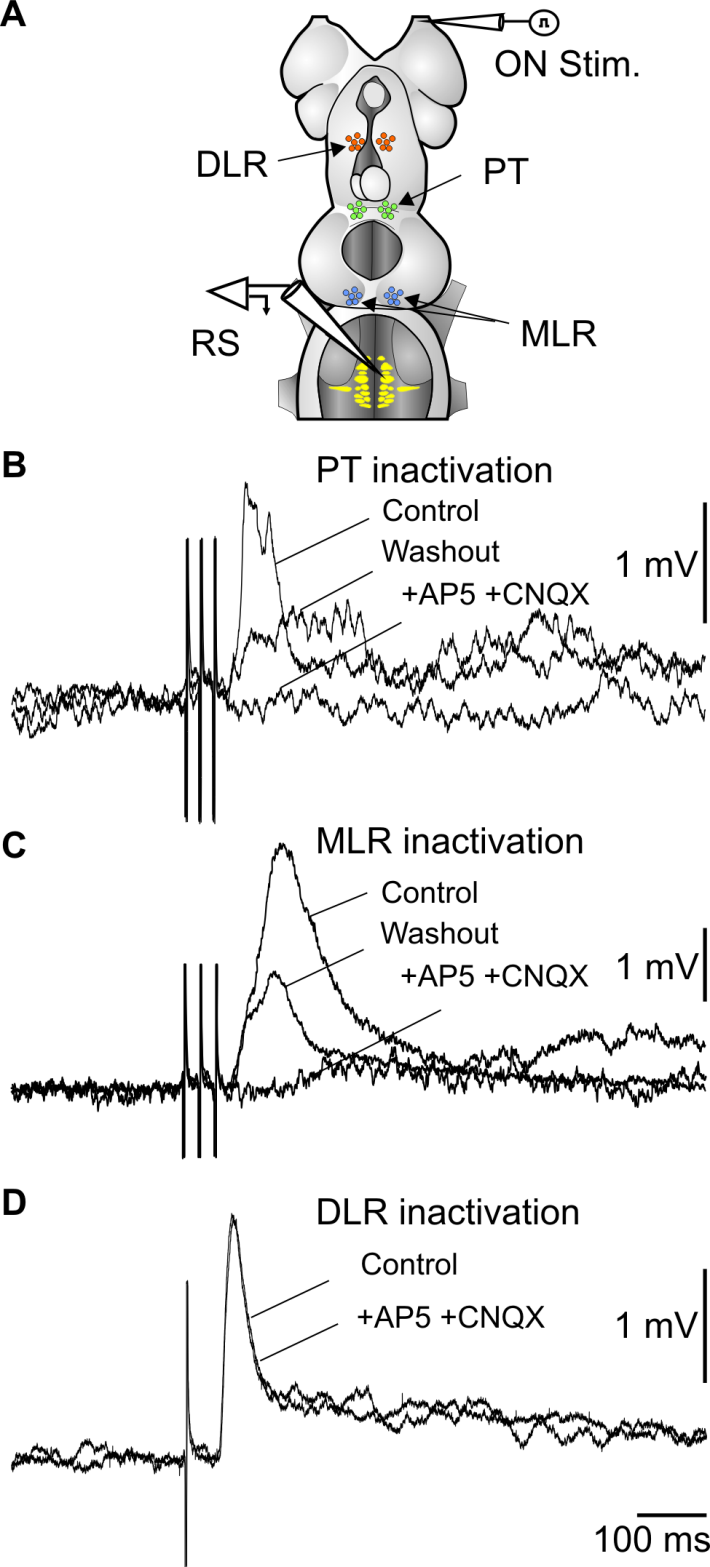
Olfactory inputs are relayed via the PT and MLR. (A) Schematic illustration showing the experimental procedure where glutamate receptor antagonists were injected in different sites indicated by the arrows. (B) RS cell responses to ON stimulation are strongly decreased by the injection in the PT. (C) Injection in the MLR has a similar effect. (D) An injection in the DLR does not block the synaptic responses. (B, C, D) are from different preparations.

We then tested the effects of stimulating the PT on RS cell activity. Single shocks (1–10 µA) elicited EPSPs in all of the RS cells tested in 12 lampreys, with a mean latency of 14±2 ms (Figure 6A). Temporal summation occurred when using trains of stimuli (5 Hz). In a semi-intact preparation (with the tail kept intact swimming freely in the recording chamber, Figure 6B), local injections of 3 mM glutamate into the PT induced swimming movements (*n* = 3, Figure 6C, D). In addition, injections of glutamate antagonists into the PT blocked the response of RS cells to ON stimulation in the isolated brain preparation (*n* = 6, Figure 7A, B). This indicates that the transmission from the medial part of the OB to the PT is glutamatergic and that the PT is indeed a key player in relaying OB inputs to locomotor centers. Because PT neurons project to the MLR [26], which in turn sends powerful inputs to RS cells [19], we investigated whether the MLR acted as an olfactory relay to RS cells. Local injections of glutamate antagonists into the MLR markedly reduced the RS response to stimulation of the ON (5–30 µA; *n* = 5, Figure 7A, C), suggesting that the MLR is involved in transmitting olfactory inputs to RS cells.

RS cells also receive projections from the DLR [22]. This region also receives inputs from the OB and is located slightly rostral and dorsal to the PT [22],[27]. A local injection of glutamate antagonists in the DLR did not block RS responses (*n* = 4, Figure 7A, D) to 5–30 µA stimulation of the ON, suggesting that DLR is not a relay in the olfactory-locomotor pathway.

## Discussion

**Figure 8:**
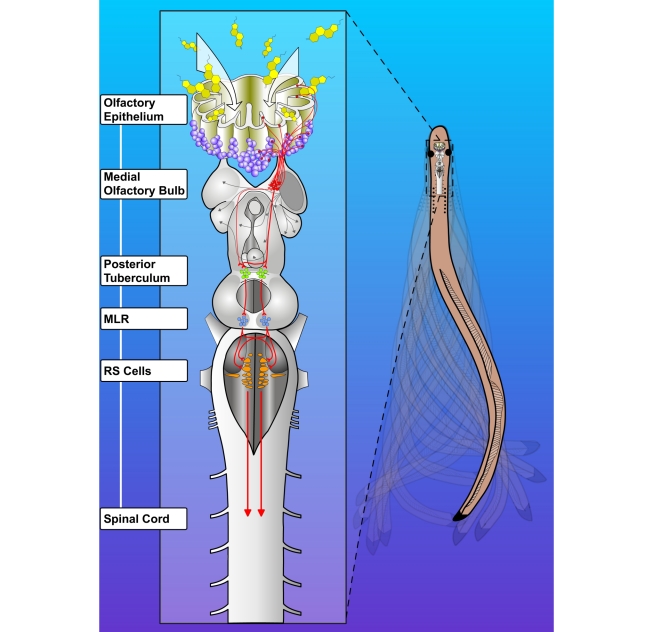
Schematic representation of the olfactory-locomotor circuitry in lampreys. Stimulation of the olfactory sensory neurons in the periphery activates neurons in the OB. There are two distinct projections from the OB, one from the lateral and another from the medial part. The lateral part projects to forebrain structures including the lateral pallium, the striatum with some fibers reaching down to habenula (grey arrows). The medial part is the relevant part for generating locomotor behavior. There is a direct projection from the medial part of the OB to the PT. From the PT, there is a projection to the MLR, known to play a crucial role in controlling locomotion in all vertebrate species. MLR neurons project to brainstem reticulospinal neurons, acting as command cells for locomotion. RS cells, in turn, project directly to spinal cord neurons that generate the basic muscle synergies responsible for propulsion during locomotion.

Sensorimotor transformations have been described in different models including lampreys [28]–[31]. Results from this study are the first description, to our knowledge, of the neural substrate underlying the transformation of olfactory inputs into a motor output in a vertebrate. This newly identified pathway originates from the medial OB and projects to the PT in the caudal diencephalon. From there, a projection to the MLR sends powerful inputs to RS cells [19] and activates spinal locomotor networks (see Figure 8). This new description of neural processing of olfactory inputs suggests that the vertebrate olfactory system could be organized in functional clusters, with a motor cluster originating from the medial OB. Interestingly, an olfactory pathway dedicated to reproduction was previously described in mammals [32]. In lampreys, there could also be other clusters related to reproduction or feeding, for example.

### Neural Substrate for the Transformation of Olfactory Inputs into Locomotion

We were able to chemically stimulate olfactory sensory neurons with naturally occurring, biologically relevant odors in a reduced in vitro preparation, and generate large excitatory responses in RS cells. These responses were elicited by different classes of odors involved in reproduction and feeding behaviors. While odorant diffusion into the nasal cavity likely accounted for RS neuron response latency, we have recently decreased the OB cell response latency to about 2 s, by applying a perfusion system for rapidly delivering larger volumes of odors within the nasal cavity by using an electronically triggered three-way solenoid valve for fast switching from normal Ringer's solution to a desired odor, without interrupting flow to the olfactory epithelium [33].

We have previously shown that sustained depolarizations in RS cells activate the spinal locomotor networks leading to swimming in lampreys [17],[18]. Thus, the elicited excitatory activity in RS cells, obtained after chemically activating olfactory sensory neurons, could generate motor activity in intact animals, such as when ovulating female lampreys are attracted by pheromones released by males [4]. In the present context, it was not possible to determine if the motor responses are attractive versus repulsive. The kinematic details of the motor output cannot be determined from ventral root recordings during fictive swimming. Further analyses in intact lampreys will be necessary to examine this.

Measurement of the latency between ON stimulation and ipsilateral RS responses provides the best indication of transmission velocity within the olfactory-locomotor pathway, as this approach was not dependent on molecular diffusion delays within the nasal cavity. We uncovered a pathway with a few synapses from the ON to RS cells. Disynaptic sensory inputs to lamprey RS cells have been well established in the past from the spinal dorsal columns [34], as well as trigeminal [35] and vestibular [36] afferents. These synaptic responses were very similar to those described here for olfactory inputs, except that we now show that four synapses are present in the olfactory-reticular pathway. As such, the first olfactory relay, the OB, does not project directly to RS cells as in other sensory systems [34]–[36], but its most caudal target is the PT [37]. In turn, RS cells do not receive inputs from structures more rostral than the diencephalon [22],[38].

The physiological and anatomical data also suggest that the medial part of the OB is necessary for eliciting motor activity in response to olfactory stimulation. Synaptic responses in RS neurons were observed only when this medial OB region was stimulated, and axons projected prominently from this site to the PT—a region with abundant dopaminergic neurons, and proposed by some to be equivalent to the ventral tegmental area of mammals [39]. We have shown that this olfactory-locomotor transformation also involves the MLR, a region with a central role in initiating and controlling locomotion [18],[19],[40]. MLR control of the power of locomotor output [18] may fine-tune swimming elicited by olfactory stimulation. While blocking the DLR did not alter the responses to olfactory inputs, the physiological context involving DLR activation remains unknown. It may have modulatory effects on olfactory-locomotor pathway under specific conditions, without directly transmitting olfactory inputs to RS cells.

### Are There Two Separate Olfactory Systems in Lampreys?

Although the organization of main olfactory epithelium and the accessory olfactory organ is not fully understood yet, recent anatomical data indicate that the accessory olfactory organ neurons project exclusively to the medial OB [11], whereas those in the main olfactory epithelium project to the entire OB. We found that canonical odorant molecules such as arginine or bile acids evoked RS cell responses very similar to those obtained with sex pheromones, and the neural pathway for the olfactory-locomotor transformation is likely to be the same in both cases. Our observations also suggest that the accessory olfactory organ only activated the locomotor pathway through the medial OB, since it does not project to more lateral OB regions. It remains to be determined whether the lateral OB receives peripheral pheromone inputs from the main olfactory epithelium. Recent observation of both odor and pheromone activation of the main and accessory OB in mice [41] has also prompted reevaluation of the classical functional separation of the olfactory system into two subsystems, with the main olfactory system responding to “odors” and the vomeronasal system responding to “pheromones.”

Our work on lampreys supports the hypothesis that the distinction between two olfactory subsystems may rely on functional output projections rather than on the olfactory molecules that first act on the system. Both anatomy and physiology show that only medial OB output neurons project to the PT, whereas other parts of the OB project to the lateral pallium. We also know that olfactory sensory neurons in both main and accessory regions of the peripheral olfactory organ project to the medial OB [11]. This suggests that the medial OB might be involved in generating motor behavior and take part in an olfactory-locomotor pathway irrespective of the type of odor molecules that are eliciting the responses. This pathway may be equally involved in food-seeking and mate-finding. The localization of olfactory transduction proteins also suggests a specific role for the medial region of the OB. The axons of olfactory sensory neurons in the medial glomerulus are not immuno-reactive to G_olf_, a cAMP-dependent olfactory G protein [12], whereas G_olf_ is localized in neurons projecting to all other parts of the OB. Accumulating evidence points to two distinct olfactory pathways in lampreys, one for specific odor detection and discrimination, and the other, the medial OB-PT pathway described here, for generating motor behavior in response to many different types of odorants.

This olfactory-locomotor transformation, through a specific pathway comprised of only a few relays in the brain, may be important for strongly linking movement responses to olfactory cues and for quickly producing motor behaviors in response to odors and pheromones. A direct projection from the OB to the diencephalon has been described in other species including fishes [38],[42],[43], amphibians [44], and mammals [45]–[47]. For instance, previous studies have reported OB projections to the PT [42],[43], the hypothalamus [45]–[47], and the habenula [44],[48]. In these vertebrates, such projections may also be important for rapid motor behaviors in response to olfactory inputs. In reptiles and mammals, the accessory OB projects to the amygdala before reaching any diencephalic region such as the hypothalamus, where these inputs likely modulate endocrine function [49]. It is noteworthy that a direct projection from the accessory OB to the hypothalamus was also seen in mammals [45]. This is particularly important with regards to the role of the hypothalamus in activating locomotion [50]. Indeed, there are several regions of the hypothalamus projecting to the MLR, and their stimulation induces stepping in mammals [50]. However, the link between olfactory inputs and locomotor responses has not been fully investigated in mammals, although locomotor behavior tightly tied to input from the olfactory system is both commonplace and remarkable. Olfactory inputs powerfully activated stepping movements in newborn rats at an early developmental stage preceding free-will locomotion [2], and locomotor responses to olfactory cues, including nipple search behavior in neonatal rabbits [51] and tracking behavior in dogs [52], were also described. In particular, recent discovery that the vomeronasal organ is directly responsible for male behavior, including vocalization, and movements associated with aggression and sexual activity in mice [53], may mean that mammals have conserved aspects of the olfactory-locomotor pathway that has been described in the present study. There could be common features as well as distinctions in olfactory-locomotor pathways in different vertebrate species. For instance, in the zebrafish, it is the lateral part of the OB that is responsible for the behavioral attraction to amino acids [54].

In conclusion, results from the present study indicate that the stimulation of the olfactory system activates the motor command system in lampreys. Locomotion is triggered by a glutamatergic pathway originating from the medial OB and projecting first to the PT, then to the MLR, before reaching RS neurons. The clear effects of physiologically blocking excitatory glutamatergic transmission to neurons in the different relays of the pathway indicate that this pathway constitutes a major component for transforming olfactory inputs into motor output. Lampreys are considered amongst the most ancestral extant vertebrate species. The conservation of such a pathway during vertebrate evolution could constitute the neural basis underlying motor responses elicited by olfactory inputs in higher vertebrates as well [2].

## Materials and Methods

### Ethics Statement

All surgical and experimental procedures conformed to the guidelines of the Canadian Council on Animal Care (CCAC) and were approved by the Université de Montréal and Université du Québec à Montréal animal care committees, and the University of Windsor animal care committee, where some of the anatomical experiments were performed.

### Animal Preparation

Experiments were performed on 98 reproductive adult sea lampreys (*Petromyzon marinus*) of both sexes provided by the Great Lakes Fishery Commission and the Department of Fisheries and Oceans Canada. In addition, some of the electrophysiology studies were carried out on larval (*n* = 62), newly transformed (*n* = 102), and parasitic (*n* = 7) lampreys. All animals were kept in aerated fresh water maintained at 7°C until used.

### Electrophysiology

For the CNS attached to the intact olfactory epithelium preparation, the animals were anesthetized with tricaine methanesulphonate (MS-222, 100 mg/l, Sigma-Aldrich, Oakville, ON, Canada) and decapitated at the level of the 7^th^ branchiopore. The surgery and all experiments were performed in cold oxygenated Ringer's (8–10°C) of the following composition (in mM): 130 NaCl, 2.1 KCl, 2.6 CaCl_2_, 1.8 MgCl_2_, 4.0 HEPES, 4.0 dextrose, and 1.0 NaHCO_3_, at pH 7.4. The branchial apparatus and the myotomal musculature were removed, with all the soft tissue attached to the ventral side of the cranium. A dorsal incision was made to expose the rostral spinal cord and the brain. The nasal cavity was left attached to the brain through the intact ONs. Care was taken to keep a maximum of the olfactory epithelium intact by simply performing a small window opening on its dorsal aspect, to simply permit faster perfusion and washout of odorants.

The preparation was placed into a recording chamber continuously perfused with cold oxygenated Ringer's at a rate of ∼4 ml/min. A minimum of 1 h was allowed for recovery after surgery prior to recording. Using sharp glass microelectrodes filled with 4 M potassium acetate (80–130 MΩ), intracellular recordings were made from RS neurons in the MRRN under visual guidance through a binocular microscope (Figure 1A). The signals were amplified with an Axoclamp 2A (Axon Instruments, Foster City, CA). Only RS neurons displaying a stable membrane potential lower than −70 mV for at least 15 min were considered in this study.

For electric stimulations, we used homemade glass-coated tungsten electrodes (4–5 MΩ with a 10 µm tip exposure) and a Grass S88 stimulator (Astro-Med, Longueuil, QC, Canada). Stimulation was applied every 10 s as single, double, or triple pulses (2–50 µA intensity, 1–2 ms duration, and 20 ms pulse interval). Synaptic responses are presented as a mean of eight consecutive responses to the same stimulation.

To observe “fictive locomotion” (Figure 3), we recorded from ventral roots in newly transformed lampreys, using suction electrodes filled with Ringer's solution. The signals were amplified using AM systems 1800 dual channel amplifiers (A-M systems Inc., Sequim, WA).

Semi-intact preparations (Figure 6B–D) were dissected as follows: the brain and rostral spinal cord were exposed like previously described [17],[19], whereas the caudal two-third of the body was kept intact to freely swim behind. In this case, because of the presence of cutaneous sensory inputs, the brain was transected at the level of the diencephalon for decerebration purposes before the experiment. The preparation was then transferred into a double compartment recording chamber. Teflon-coated stainless steel microwires (50 µm diameter) were inserted into the segmental muscles for EMG recording.

### Calcium Imaging Experiments

RS cells were retrogradely labeled in Ringer's solution for 24 to 36 h by placing Calcium-Green dextran crystals (3000 MW, Invitrogen, Eugene, OR) on the rostral stump of the spinal cord, transected at the first segment. Labeled cells were observed on a Nikon epifluorescent microscope equipped with a 20× (0.75 NA) objective. A fluorescein isothiocyanate (FITC) excitation/emission filter set was used to visualize the neurons. The emitted light was captured with an intensified CCD video camera (Photometrics CoolSNAP HQ, Roper Scientific, Tucson, AZ) and recorded at a rate of two images per second, using Metafluor imaging software (Molecular Devices, Sunnyvale, CA). Calcium responses are expressed as relative changes in fluorescence (ΔF/F%).

### Anatomical Experiments

Anterograde and retrograde labeling was obtained after unilateral injections of Texas Red-conjugated dextran amines (3000 MW, Molecular Probe). A period of 24 to 36 h was allowed for the transport of the tracer. The preparations were then immersed in a solution of 4% paraformaldehyde/0.4% picric acid in phosphate-buffered saline for 5 to 6 h. They were then transferred overnight into a solution of 20% sucrose in phosphate buffer. Transverse sections of 25 µm thickness were made with a cryostat and mounted on microscope slides with Vectashield (Vector laboratories, Burlington, ON, Canada) for observation under epifluorescence microscopy (Nikon E600 microscope equipped with a DXM1200 digital camera, Nikon, Montreal, QC, Canada; or with a Nikon E800 microscope in Windsor, ON, Canada).

### Chemical Stimulations and Drugs

All drugs were purchased from Sigma-Aldrich (Oakville, ON, Canada), except sex pheromones (graciously provided by Dr. W. Li, Michigan State University, MI). They were kept as frozen concentrated stock solutions (at −80°C for pheromones) and dissolved to their final concentration in Ringer's solution prior to their use. For all local ejections, the inactive dye Fast Green was added to the drug solution to monitor the extent of application. Drug application was performed by pressure ejections of concentrated substances through a glass micropipette in the nasal cavity, or the brain tissue, using a Picospritzer (General Valve, Fairfield, NJ). Ejection of Ringer's with Fast Green at the same location was used as control in each experiment. Chemical stimulations of the olfactory epithelium were performed on reproductive adult lampreys during the first days after their capture in the wild.

### Statistics

Data were analyzed using paired *t* test or a Mann-Whitney test (Sigmastat, SPSS, Chicago, IL, USA). Significance was set at *p*<0.05. Results are presented as mean ± S.E.M.

## Abbreviations

CNS: central nervous system
DLR: diencephalic locomotor region
MLR: mesencephalic locomotor region
MRRN: middle rhombencephalic reticular nucleus
OB: olfactory bulb
ON: olfactory nerve
PT: posterior tuberculum
RS: reticulospinal
